# Facet-selective morphology-controlled remote epitaxy of ZnO microcrystals via wet chemical synthesis

**DOI:** 10.1038/s41598-021-02222-1

**Published:** 2021-11-22

**Authors:** Joonghoon Choi, Dae Kwon Jin, Junseok Jeong, Bong Kyun Kang, Woo Seok Yang, Asad Ali, Jinkyoung Yoo, Moon J. Kim, Gyu-Chul Yi, Young Joon Hong

**Affiliations:** 1 Department of Nanotechnology and Advanced Materials Engineering, Sejong University, Seoul, 05006 Republic of Korea; 2 GRI–TPC International Research Center, Sejong University, Seoul, 05006 Republic of Korea; 3 Nano Materials Research Center, Korea Electronics Technology Institute (KETI), Seongnam, Gyeonggi-do 13509 Republic of Korea; 4 Department of Electronic Materials and Devices Engineering, Department of Display Materials Engineering, Soonchunhyang University, Asan, Chungnam 31538 Republic of Korea; 5 Department of Physics and Astronomy, Institute of Applied Physics, Seoul National University, Seoul, 151-747 Republic of Korea; 6 Center for Integrated Nanotechnologies (CINT), Los Alamos National Laboratory, Los Alamos, NM 87545 USA; 7 Department of Materials Science & Engineering, The University of Texas at Dallas, Richardson, TX 75080 USA

**Keywords:** Graphene, Nanowires, Synthesis and processing, Nanowires

## Abstract

We report on morphology-controlled remote epitaxy via hydrothermal growth of ZnO micro- and nanostructure crystals on graphene-coated GaN substrate. The morphology control is achieved to grow diverse morphologies of ZnO from nanowire to microdisk by changing additives of wet chemical solution at a fixed nutrient concentration. Although the growth of ZnO is carried out on poly-domain graphene-coated GaN substrate, the direction of hexagonal sidewall facet of ZnO is homogeneous over the whole ZnO-grown area on graphene/GaN because of strong remote epitaxial relation between ZnO and GaN across graphene. Atomic-resolution transmission electron microscopy corroborates the remote epitaxial relation. The non-covalent interface is applied to mechanically lift off the overlayer of ZnO crystals via a thermal release tape. The mechanism of facet-selective morphology control of ZnO is discussed in terms of electrostatic interaction between nutrient solution and facet surface passivated with functional groups derived from the chemical additives.

## Introduction

Epitaxy is an ideal method for growing single crystal semiconductor device components on other wafers, and the technique has demonstrated numerous high-performance practical solid-state electronic and optoelectronic devices (e.g., high electron mobility transistors, laser diodes, light-emitting diodes, single photon emitters/detectors, etc.)^1,2^. Recently, non-covalent epitaxy (i.e., van der Waals epitaxy and remote epitaxy) of semiconductors on two-dimensional (2d) van der Waals (vdW) atomic layers^3–5^ has gained considerable interest because of the ability of creating releasable devices based on slippery surface feature of 2d vdW substrates^3,6,7^. Distinct from the vdW epitaxy, the remote epitaxy showed a great potential for practical high performance device fabrications and applications^8,9^ because the single crystalline overlayers are easily obtained despite multi-domain of the 2d gap layer (e.g., graphene)^3,10,11^. This lattice transparency has so far allowed to expand the growing materials to binary and ternary compound semiconductors, metal oxide semiconductors, and halide perovskite in forms of single-crystalline thin film^3,12–14^ or uni-directionally aligned microrods^8,11,15^. Then, the remote epitaxial overlayers were successfully delaminated via adhesive tape exfoliation technique without chemical etching and high energy irradiation melting (e.g., laser lift-off) of sacrificial layer^13,16,17^. For variety of practical applications, the remote epitaxy and release process are desirable to expand to diverse growth techniques and overlayer morphologies.

In the bottom-up approach for fabricating non-planar overlayer, morphological control is essential to apply them to functional devices because size, shape, and geometry of the as-synthesized semiconductor crystal overlayers determine the physical properties and device performances (e.g., carrier confinement, mobility, laser Q factor, light extraction efficiency, energy storage capacity, depletion geometry at the junction of nanostructure/substrate, etc.)^18–25^. Moreover, the control of morphology offers an opportunity to determine the dominant surface facets of crystals that are associated with surface chemical properties of photocatalytic, water-splitting efficiencies, anti-microbial activity, adsorption properties, and so on^26–32^. In the wet chemical synthesis technique, several representative approaches have been developed for control of morphology and facet selective anisotropic growth^19,33^. For example, surfactant type-capping agent was used for controlling the coordination of precursor^34^; precursor molar ratio is also critical in inhibiting the growth of specific crystal planes, which leads to control the shape of crystals^35,36^; and pH governs the dissolution–reprecipitation of precursors for size and facet control^35^. Despite the importance and diverse successful methods of morphological control, it has been rarely reported how the morphology of overlayer is controlled in the non-covalent epitaxy. Since the remote epitaxy is driven by the potential (or polarity field) penetrated across gap layer of graphene from the underlying wafer, the adsorption energy on graphene-coated wafer is weaker than that for ionic or covalent epitaxy directly on bare wafer. The weakened surface electrostatic potential on graphene changes the size of critical nuclei and growth density^14^. For the reason, the nucleation–growth of remote epitaxy for the morphology control is a challenge and prerequisite toward the above-mentioned applications.

ZnO is a wide direct bandgap semiconductor (3.3 eV) with high exciton binding energy (60 meV)^37^, and the bandgap can be modulated in a wide range by alloying with Cd and Mg^38^. Due to the excellent optical properties, ZnO-based heterojunctions have been applied to light-emissive and photovoltaic devices^22,39–41^. Furthermore, the highly non-centrosymmetric crystal structure of ZnO was utilized as high efficiency piezoelectric generator in a form of nanowire^42–44^, and such high polarity enabled the remote homo- and heteroepitaxy via the hydrothermal growth^11,45–47^. Due to the potential practical applications, it is desirable to control the morphology of ZnO crystals through the remote epitaxy. Herein we report the method of controlling morphology of ZnO micro- and nanostructures via hydrothermal synthesis-based remote epitaxy. The facet-selective morphology is controlled by changing the additives of nutrient solution. We discuss the mechanism of morphology control in terms of facet-dependent growth rate determined by electrostatic interaction between nutrient solution and facet surface passivated with functional groups derived from the chemical additives. Transmission electron microscopy corroborates the remote epitaxial relation between ZnO and GaN substrates across graphene. Raman spectroscopy confirms the existence of the graphene basal layer after the hydrothermal growth of ZnO. The ability of remote epitaxy to mass-release of ZnO microarrays from the original substrate is demonstrated, based on a sticky tape delamination technique.

## Methods

### Substrate preparation and wet chemical remote epitaxy of ZnO

The remote epitaxy of ZnO was carried out on graphene-coated GaN(0001) substrate through hydrothermal synthesis using zinc nitrate hexahydrate (ZNH, Zn(NO_3_)_2_·6H_2_O, 25.0 mM) and hexamethylenetetramine (HMTA, C_6_H_12_N_4_, 25.0 mM). For controlling the morphology of ZnO, an additive of hydrochloric acid (8.8 mM), trisodium citrate dihydrate (TCD, Na_3_C_6_H_5_O_7_, 0.34 mM), or zinc acetate dihydrate (ZAD, Zn(CH_3_CO_2_)_2_·2H_2_O, 25.0 mM) was used so that growth of specific crystal plane was either facilitated or inhibited for facet selective anisotropic growth. The growth was performed at 95 °C for 4 h in a Teflon-lined autoclave. For preparing the remote epitaxy substrate, single-layer graphene (SLG) was grown on copper foil by chemical vapor deposition (CVD)^48^, then was transferred onto *c*-plane GaN/Al_2_O_3_ wafer by the poly(methyl methacrylate) (PMMA)-supported wet etching–transfer technique.

### Exfoliation of ZnO overlayer from substrate

Before the exfoliation of ZnO overlayer structures, gaps between ZnO crystals were filled by spin-coating of polyimide (PI). The PI encapsulating layer was then dried at 120 °C for 2 min, and cured at 300 °C for 5 min. After the coating process, the film of PI-encapsulated ZnO crystals was peeled off from the donor substrate of GaN/Al_2_O_3_ using a thermal release tape by a handwork. This encapsulating layer structurally supports the as-grown arrangement of ZnO crystals without breaking the as-grown deployment during the exfoliation (or transfer).

### Characterizations

The surface morphologies of remote epitaxial ZnO crystals were observed by field-emission scanning electron microscopy (FE-SEM, Hitachi S-4700). The crystal structures and remote epitaxial relation of the samples were scrutinized using atomic-resolution scanning transmission electron microscopy (AR-STEM, Jeol JEM-ARM 200F) and the selective area electron diffraction (SAED) analyses. For the AR-STEM observations, the samples were cross-sectionally milled by a 10–30 kV-accelerated beam of gallium ions in a focused ion beam machine (FIB, FEI Nova 2000) for the bulk milling, and 5 kV-low energy milling was introduced to reduce microstructural damage on the sample. The incidence electron beam was directed along [$\overline{1}2\overline{1}0$] to corroborate the wurtzite crystal structures of the remote heteroepitaxial ZnO(0001)/GaN(0001) in the STEM analysis. The convergence semi-angle and the current of the electron probe were set to 24 mrad and 23 pA, respectively. The collection semi-angle in the high-angle annular dark-field (HAADF) and annular bright field (ABF) imaging was 70–250 mrad and 12–24 mrad, respectively. The optical properties of micro- and nanostructure ZnO crystals were investigated by using photoluminescence (PL) spectroscopy with excitation light of 325 nm wavelength He–Cd laser (25 mW, Kimmon IK3201R-F) and a monochromator (AndorDU401A-UV) with a photomultiplier system. The temperature-dependent and low-temperature PL spectra were obtained at 12–120 K in cryopump-based closed cycle hellium refrigeration system. The presence of graphene after the hydrothermal growth and exfoliation process was also investigated by Raman spectroscopy (Renishaw 2000) with 514 nm wavelength-excitation laser (20 mW).

## Results and discussion

### Morphology-controlled remote epitaxy

**Figure 1:**
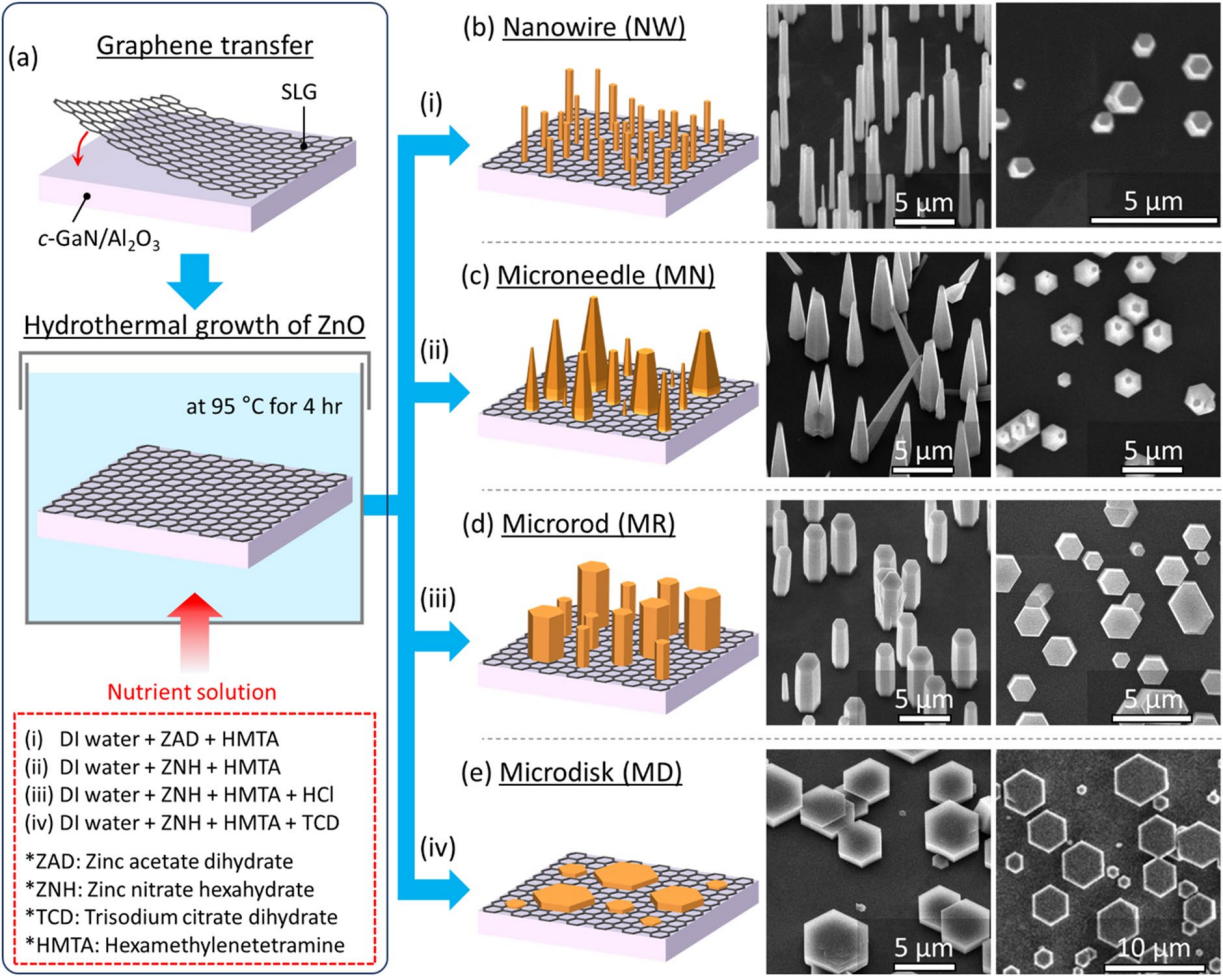
Morphology-controlled hydrothermal remote heteroepitaxy of ZnO micro- and nanostructures on GaN substrate through graphene. (**a**) Illustration depicting procedures of hydrothermal remote epitaxy of ZnO on graphene-coated *c*-GaN substrate. (**b**)–(**e**) Schematic and SEM images of ZnO nanowire (NW), microneedle (MN), microrod (MR), and microdisk (MD) arrays obtained from nutrient solutions of (i), (ii), (iii), and (iv), respectively, described with abbreviated chemical precursors in red box in (**a**).

Remote heteroepitaxial growth of ZnO was begun with transfer of the CVD-grown SLG onto GaN/Al_2_O_3_(0001) wafer through the PMMA-supported etching–transfer technique. Then, ZnO micro- and nanostructured crystals were hydrothermally grown on the SLG-coated GaN substrate in four different nutrient solutions, described in (i)–(iv) of Fig. 1a, at 95 °C for 4 h. Those wet chemical solutions yielded four distinct crystal morphologies, which were nanowires (NWs), microneedles (MNs), microrods (MRs), and microdisks (MDs), as displayed in Fig. 1b–e. Without the use of TCD additive, ZnO NWs, MNs, and MRs with high height-to-diameter (*h*/*d*) ratios were grown to exhibit good vertical alignment (Fig. 1b–d). When the TCD was introduced in the nutrient solution, ZnO was grown to a hexagonal platelet shape. Noticeably, all these ZnO crystals exhibited a uniform in-plane alignment of hexagonal sidewall facets over the whole substrate area. Note that the CVD-grown SLG had a typical domain size of 5–20 μm (Fig. S1). If the carbon lattice of SLG ruled the epitaxial relation, the uniform in-plane alignment should have been limited within each domain^45^. For the reason, the epitaxial relation is likely originated from the underlying single-crystalline GaN across ultrathin SLG.

In this study, there were no clear evidences that microcrystals are preferably or rarely formed along domain boundaries of CVD-grown graphene, distinct from the preferential growth of nanostructures along the step edges of exfoliated graphene^49,50^. The small-angle domain boundaries with few dangling bonds and steps hardly caused the preferential growth in the remote epitaxy, and we believe that polarity penetration across graphene and potential energy fluctuation on graphene played more impactful role in nucleation and growth of remote-epitaxial ZnO microcrystals^8,51^.

**Figure 2:**
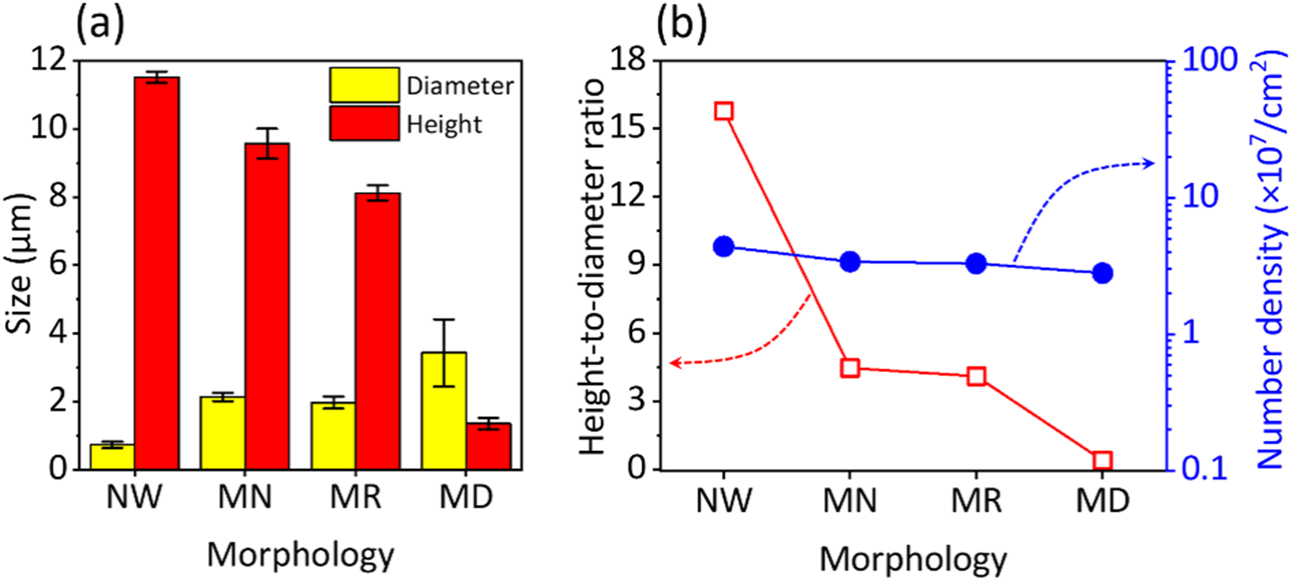
Morphological information of ZnO grown by the remote epitaxy. (**a**) Mean diameter (yellow columns) and height (red columns) values. The diameter of NW, MN, MR, and MD was measured at 0.73 ± 0.09, 2.14 ± 0.12, 1.98 ± 0.18, and 3.44 ± 0.99 µm, respectively; the height of those was at 11.52 ± 0.17, 9.57 ± 0.44, 8.13 ± 0.22, and 1.36 ± 0.17 µm. Error bars represent ± standard deviation values. (**b**) *h*/*d* ratios (red empty squares) and number density (blue filled circles) for different ZnO morphologies.

To quantify the differences in morphology of ZnO crystals, the diameter, height, *h*/*d* ratio, and number density of ZnO were measured, as summarized in Fig. 2. As the morphology was changed from NW to MD, the mean height was drastically decreased from 11.5 ± 0.2 to 1.4 ± 0.2 μm, whereas the mean diameter of ZnO crystals was increased from 0.7 ± 0.1 to 3.4 ± 1.0 μm (Fig. 2a). Thus, the *h*/*d* ratio was calculated to decrease from 15.8 to 0.4 for the morphological change. Since the concentration of zinc-containing precursors was same at 25.0 mM for growing those crystals, the number density values were not significantly varied (ca. 3 × 10^7^ cm^–2^), irrespective of ZnO morphology (blue solid circles of Fig. 2b). As the concentration of equimolar nutrient solution was diluted, the size and density of microcrystals were reduced without morphology variations (Fig. S2). Accordingly, the amount of supplied precursor is responsible for the change of microcrystal size, and we surmise that the additives led to facet-dependent anisotropic growth by causing different electrostatic interactions between aqueous additives and charged surface of ZnO facets.

### Synthetic strategy for facet-selective morphology control

In the hydrothermal nutrient solution, zinc cations (Zn^2+^) are supplied from ZNH (or ZAD), while hydroxide anions (OH^–^) from HMTA, according to the chemical Eqs. (1) and (2). Then, the zincate ions (Zn(OH)_4_^2–^) are formed from Zn^2+^ and OH^–^ (Eq. (3)). Eventually, the zincate ions decompose to form ZnO crystals, and by-products of H_2_O and OH^–^ are generated, simultaneously (Eq. (4))^52^.

1 $$\left( {{\text{CH}}_{{2}} } \right)_{{6}} {\text{N}}_{{4}} + {\text{ H}}_{{2}} {\text{O }} \to {\text{ 4NH}}_{{3}} + {\text{ 6HCHO}}$$

2 $${\text{NH}}_{{3}} + {\text{ H}}_{{2}} {\text{O }} \to {\text{ NH}}_{{4}}^{ + } + {\text{ OH}}^{-}$$

3 $${\text{Zn}}^{{{2} + }} + {\text{ 4OH}}^{-} \to {\text{ Zn}}\left( {{\text{OH}}} \right)_{{4}}^{{{2}{-}}}$$

4 $${\text{Zn}}\left( {{\text{OH}}} \right)_{{4}}^{{{2}{-}}} \to {\text{ ZnO }} + {\text{ H}}_{{2}} {\text{O }} + {\text{ 2OH}}^{-}$$

**Figure 3:**
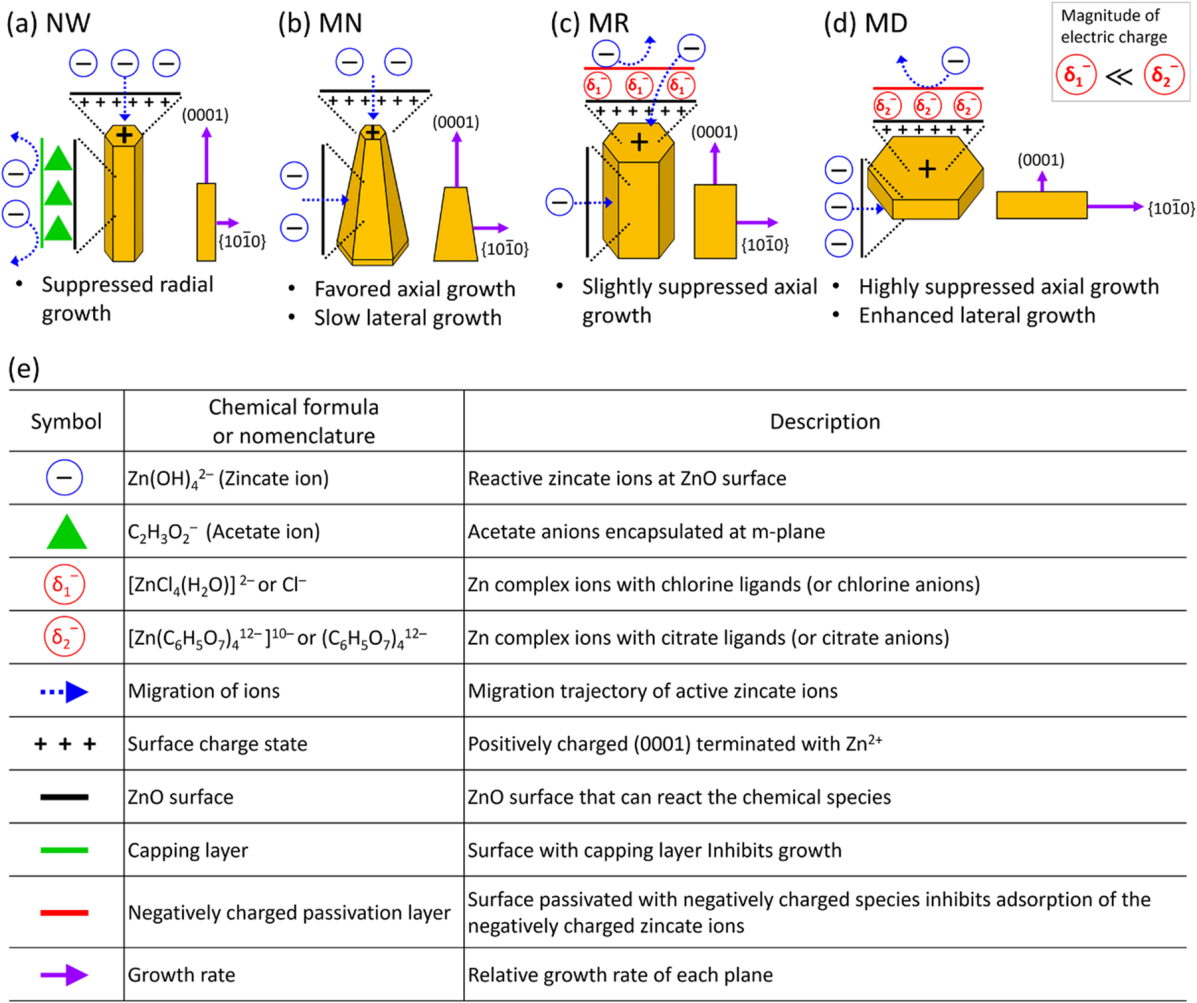
Facet-selective electrostatic model for morphology-controlled remote epitaxy of ZnO crystals. Illustrations depicting facet-dependent inhibited (or enhanced) growth for ZnO (**a**) NW, (**b**) MN, (**c**) MR, and (**d**) MD. (**e**) Summarized table describing symbols and those roles in the morphology control.

Figure 3a–d illustrates the growth procedures how the NW, MN, MR, and MD structures are selectively formed, based on the electrostatic interaction between zincate ions and the surface charge of ion-capped ZnO facets by the nutrient solution with different additives. The roles of ions and symbols schematized in Fig. 3a–d are described in table of Fig. 3e. The NWs with high *h*/*d* ratios were typically obtained in the solution with ZAD and HMTA nutrients. The use of the ZAD reagent forms acetate anions (C_2_H_3_O_2_^–^) that bring a sidewall capping effect, which substantially limits the radial growth, as depicted in Fig. 3a. Then, the screened zincate anions migrate along the NW sidewall, and are preferably adhered onto Zn^2+^-terminated (0001) top of NW because of strong Coulombic attraction. This leads to rapid anisotropic *c*-axial growth of NW for high *h*/*d* ratio. It is well known that the surface energy is determined by the species of ion in the nutrient solution. Noticeably, Qin et al. reported that the surface energy of sidewall m-plane, simulated via the density functional theory (DFT), is lower than Zn-polar c-plane of ZnO by ca. 1.0 eV in the solution with acetate anions because the acetate ions are electrostatically adsorbed to the sidewall m-plane for the capping effect^53^. The simulated results well explain the rapid growth along c-axis with the suppressed sidewall radial growth in our empirical study.

When the ZAD was replaced by ZNH, the nutrient solution of (ZNH + HMTA) resulted in formation of tapered MN morphology rather than NW (solution (ii) of Figs. 1c and 3b). Negatively charged zincate ions were favored to adhere onto positively charged (0001) top of MN terminated with Zn^2+^, and a radial growth also occurred. The sidewall capping effect was not so much strong in comparison with the growth of NWs, thus the slow radial growth resulted in the tower-shape tapered MN structure with low sidewall slant angle. It is plausible that stronger interaction between MN topside and zincate anions caused higher longitudinal growth rate than the radial one. The angle of MN sidewall was measured at 87.8 ± 0.9°, much steeper than that of {$10\overline{1}1$} slants (≈ 57.4°) in the typical hexagonal pyramid. The nearly upright MN sidewall, corresponding to higher index {$h0\overline{i}1$}, consisted of the layer-by-layer multiple steps as observed by high magnification SEM imaging (Fig. S3). The mechanism of multi-step formation has been discussed by the anisotropic growth rate in layer-by-layer growth. Before completing the growth of the bottom layer, new layers of (0001) plane are nucleated and grown in the layer-by-layer growth, and the bottom layer still grow radially. This presumably caused the formation of multi-steps on the MN sidewalls^54,55^.

**Figure 4:**
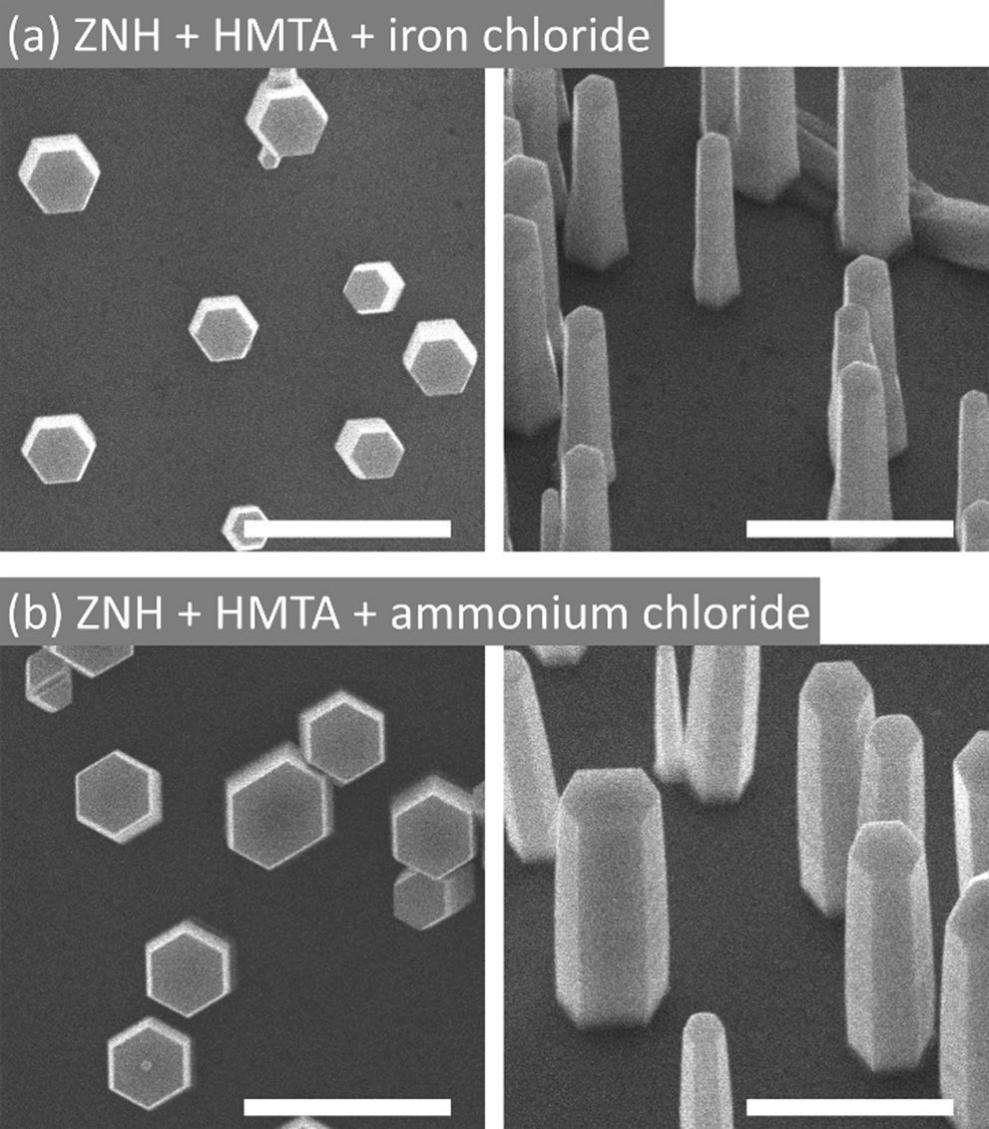
SEM images of ZnO MRs grown by using different chloride additives. Top- and tilt-view SEM images of MRs grown in the nutrient solution of ZNH and HMTA with an additive of (**a**) iron chloride and (**b**) ammonium chloride. The same nutrient solutions with chloride additives of an equimolar concentration of 8.8 mM were used for both (**a**) and (**b**). All scale bars are 5 μm.

**Figure 5:**
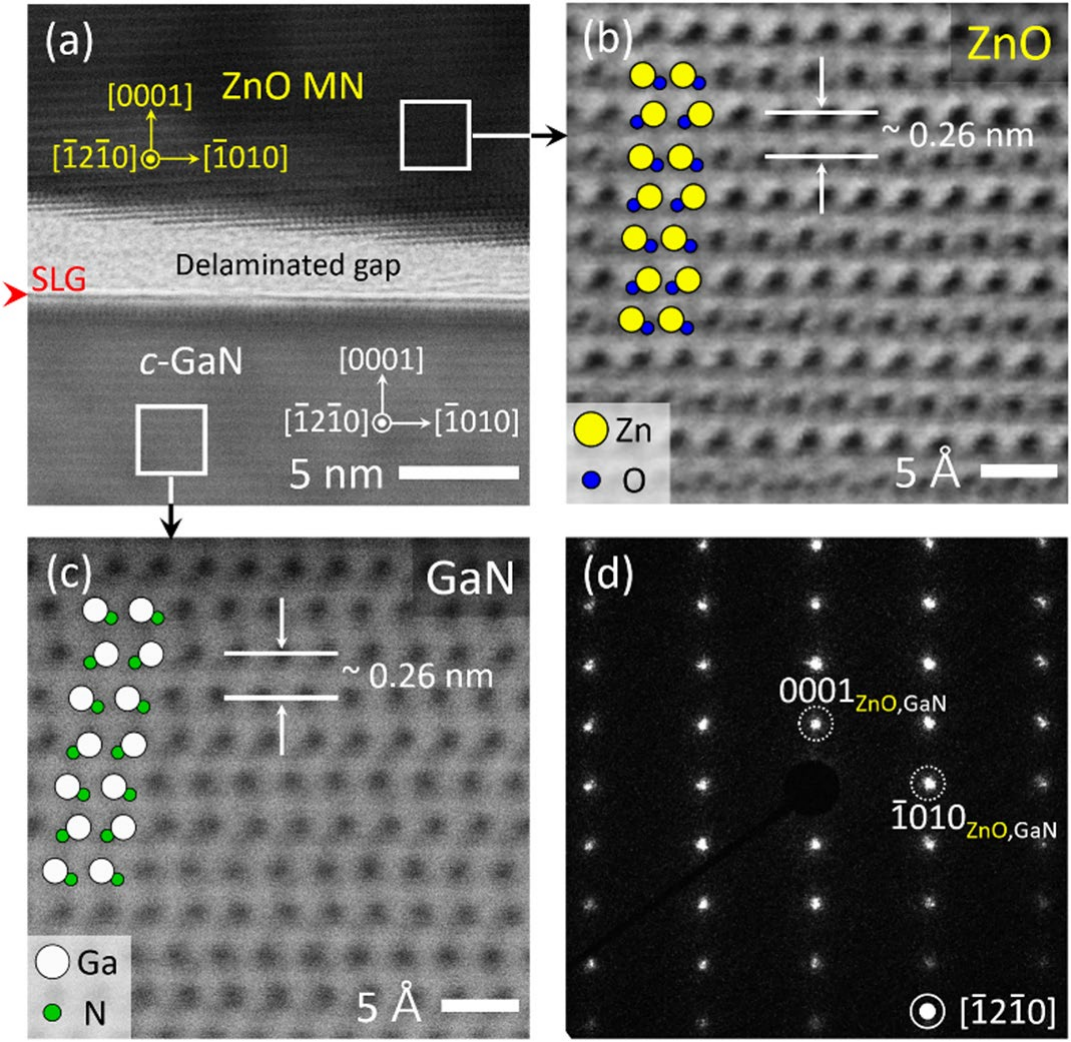
Cross-sectional AR-STEM and SAED analyses of remote epitaxial ZnO MN grown on SLG/GaN. (**a**) Low magnification image of *c*-ZnO MN/SLG/*c*-GaN. AR-STEM image of (**b**) ZnO MN and (**c**) *c*-GaN substrate in a bright-field mode, taken from boxed areas marked in (**a**). (**d**) SAED patterns of the remote epitaxial c-ZnO MN/SLG/c-GaN.

By adding HCl in the (ZNH + HMTA) solution, the morphology was changed to non-tapered MRs. The addition of HCl induced the formation of both zinc complex ion having chlorine ligands [ZnCl_4_(H_2_O)]^2–^ and Cl^–^ (Refs.^56,57^). The Zn-terminated ZnO *c*-plane is slightly screened by adsorption of [ZnCl_4_(H_2_O)]^2–^ or Cl^–^ because of the Coulombic attraction with positively charged Zn-terminated (0001) top (Fig. 3c), and the slightly screened *c*-plane surface is thought to be responsible for the longitudinal growth rate slower than MNs (Fig. 2a)^58^. It is observed that our TEM monitoring showed the Zn-terminated *c*-plane (Fig. 5b). Interestingly, the DFT simulation work by Qin et al. predicted that lower chlorine anion makes the Zn-terminated (0001) stable while the m-plane becomes more activated than the c-plane for radial growth^53^. We surmise that the zincate ions preferred to binding with the unstable m-plane for a substantial total energy minimization, which contributed to the radial growth for forming disk-shaped ZnO. We note that the role of chlorine additive in forming non-tapered MRs was also validated by adding different additives of ion chloride and ammonium chloride, as shown in Fig. 4a,b, respectively. However, the use of too much HCl etched away or left a harsh damage on ZnO MRs (Fig. S4).

The use of TCD additive in the (ZNH + HMTA) induced highly suppressed axial growth of MDs (Fig. 3d). The TCD led to formation of [Zn(C_6_H_5_O_7_)_4_^12–^]^10–^ and citrate anions of (C_6_H_5_O_7_)_4_^12–^ (Ref.^59^). Because of highly negative charge of [Zn(C_6_H_5_O_7_)_4_^12–^]^10–^, the *c*-plane ZnO can be well passivated by the citrate ligands so that zincate ions are hardly adhered onto top *c*-plane. Instead, the zincate ions contributed to the high lateral growth rate yielding MDs^27,60^.

The growths of MN, MD, MR, and NW were reliably reproducible over again, owing to the use of precursors and additives that can electrostatically interact with specific crystal planes so that enabled to easily achieve each anisotropic growth. Among the morphologies, the synthesis of MR was slightly sensitive to reproduce than the other structures. To reliably obtain the thick rod morphology, it is essential to control the growth rates of *c*- and *m*-planes similar for yielding *d*/*h* ratio of ca. 4. This was delicately controlled at acidic pH condition of 5.5–5.8. The reproducibility test results for MRs are displayed in Fig. S5.

### Transmission electron microscopic analysis

Crystal structure and the remote heteroepitaxial relationship of *c*-ZnO MN/SLG/*c*-GaN were characterized by AR-STEM analysis. A cross-sectional TEM image of *c*-ZnO MN/SLG/*c*-GaN heterointerface shows the presence of SLG on top of *c*-GaN, as marked with a red wedge in Fig. 5a. This indicates that the wet chemical condition in this study did not etch away graphene. During the milling process, a slight gap between SLG and GaN was opened because the loosely bound, non-covalent interface facilitated delamination even under a low energy milling condition. In the AR-STEM image, the ZnO MN and GaN substrate exhibited close-packed ABAB stack sequence of wurtzite along Zn-terminated (or O-initiated) and Ga-terminated < 0001 > direction, respectively (Fig. 5b,c). This observation indicates that the polarity inversion did not occur despite insertion of graphene gap layer in between two WZ structures. Interplanar spacings were measured at 0.26 nm at both sides, corresponding to *d*_0002_ of wurtzite structure of ZnO and GaN. In the SAED analysis, the regular diffraction spots were obtained without irregular spots caused by polycrystallinity or non-epitaxial relation (Fig. 5d). The AR-STEM analysis indicated the remote epitaxial relationship of (0001)[$10\overline{1}0$]_ZnO_∥SLG∥(0001)[$10\overline{1}0$]_GaN_.

### Photoluminescence characterization

**Figure 6:**
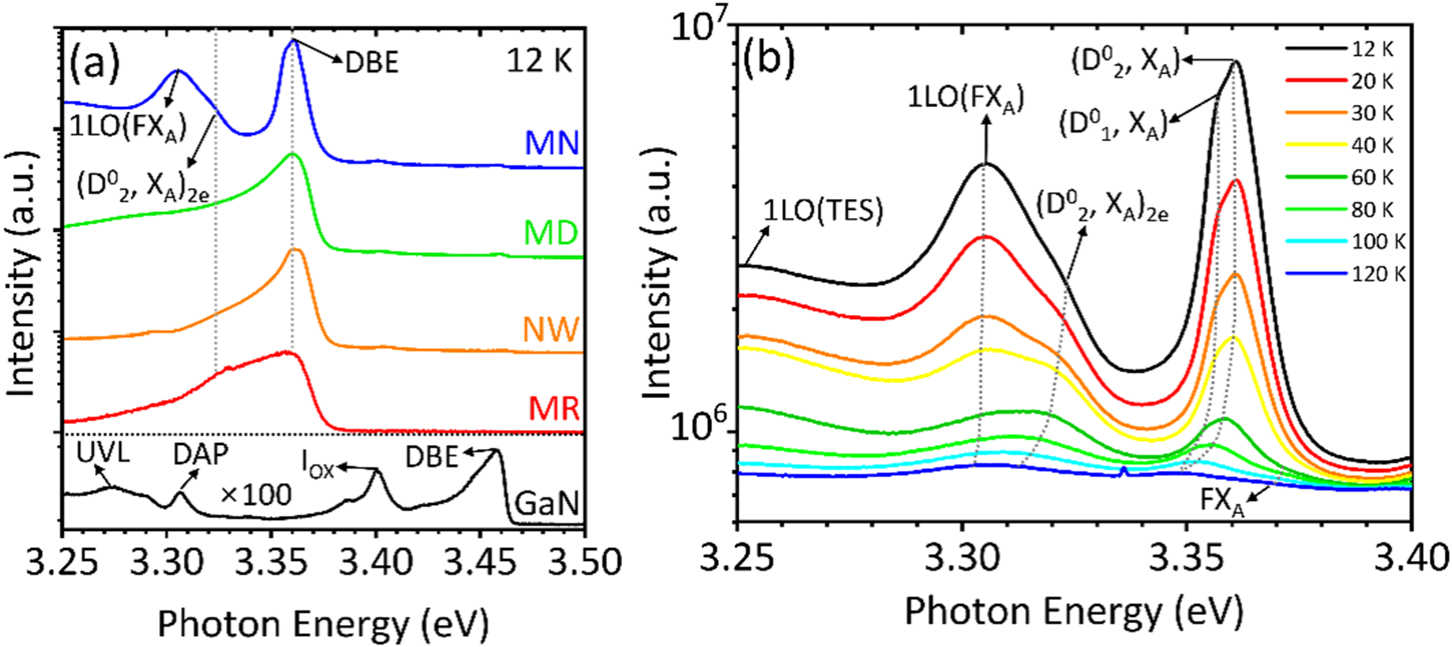
Optical properties of remote epitaxial ZnO crystals/SLG/GaN. (**a**) Low-temperature PL spectra of remote epitaxial ZnO crystals. (**b**) Temperature-dependent PL spectra of MN. a.u. denotes arbitrary units.

Optical properties of the ZnO micro- and nanostructures were characterized by measuring PL spectra at low temperature. Figure 6a shows 12 K PL spectra of the remote epitaxial ZnO crystals grown on SLG/GaN substrate. The low temperature PL spectrum of bare GaN showed four distinct excitonic emission peaks attributable to neutral-donor bound exciton (DBE), oxygen-related luminescence (I_ox_), donor–acceptor-pair (DAP) transition, and ultraviolet (UVL) band at 3.458, 3.400, 3.305, and 3.26–3.30 eV, respectively^61,62^. All the PL spectra of ZnO crystals grown on SLG/GaN exhibited a dominant PL peak at 3.360 eV, ascribed to DBE of ZnO, denoted as (D^0^, X)^37^. It should be noted that carbon-associated PL peak at 3.356 eV, originating from extrinsic substitutional donor (C_Zn_), was not clearly observed as neutral acceptor bound exciton. This suggests that carbon from graphene was not incorporated into ZnO during the hydrothermal growth for high purity of ZnO^49,63^, and the observation of the strong near band edge (NBE) emission also indicates high optical quality of remote epitaxial ZnO microcrystals. Two-electron satellite (TES) PL emissions, denoted as (D^0^_2_, X_A_)_2e_, were observed as shoulder at 3.321 eV from all the ZnO samples, and especially the MNs clearly showed longitudinal optical mode of free exciton, denoted with 1LO(FX_A_), at 3.305 eV and 1LO mode of TES at around 3.25 eV at 12 K (Fig. 6b)^64,65^. The different PL spectral shapes of ZnO crystals are tentatively attributable to the use of different nutrients and additives.

Among the samples, the PL property of MNs was further characterized at diverse temperatures. The high-resolution temperature-dependent PL spectra of Fig. 6b display well-resolved DBE peaks consisted of (D^0^_2_, X_A_), (D^0^_1_, X_A_), and (D^0^_2_, X_A_)_2e_ at 3.360, 3.358, and 3.321 eV, respectively, at 12 K^66^. Importantly, as marked in Fig. 6b (see also Fig. S6), free exciton (FX_A_) transition was observed as a shoulder peak at 3.370–3.372 eV above 60 K in the vicinity of the DBE band, and the 1LO(FX_A_) peak was also clearly observed at 3.302–3.305 eV at various temperatures. In the temperature-dependent PL measurement, the quenching of 1LO(FX_A_) was less than that of DBE peaks, which is an evidence of free exciton-relevant PL emission^65^. The observations of the FX_A_ and the relevant PL peaks suggest high optical quality of MNs grown by the hydrothermal remote epitaxy. Among the samples, only the MNs clearly exhibited (i) well-resolved NBE transition PL peaks and (ii) the free exciton PL peaks (Fig. 6b and Fig. S6). As listed in Tables S1 and S2 (see Supplementary Materials), the use of high purity precursors without additives is likely the main reason for the two PL signatures of the MNs. However, though the growth of NWs employed high purity nutrient solution without the use of additives, such as TCD and HCl, the NWs did not exhibit the high optical quality, presumably due to high surface-to-volume ratio.

### Microcrystal overlayer delamination and Raman spectroscopic analysis

**Figure 7:**
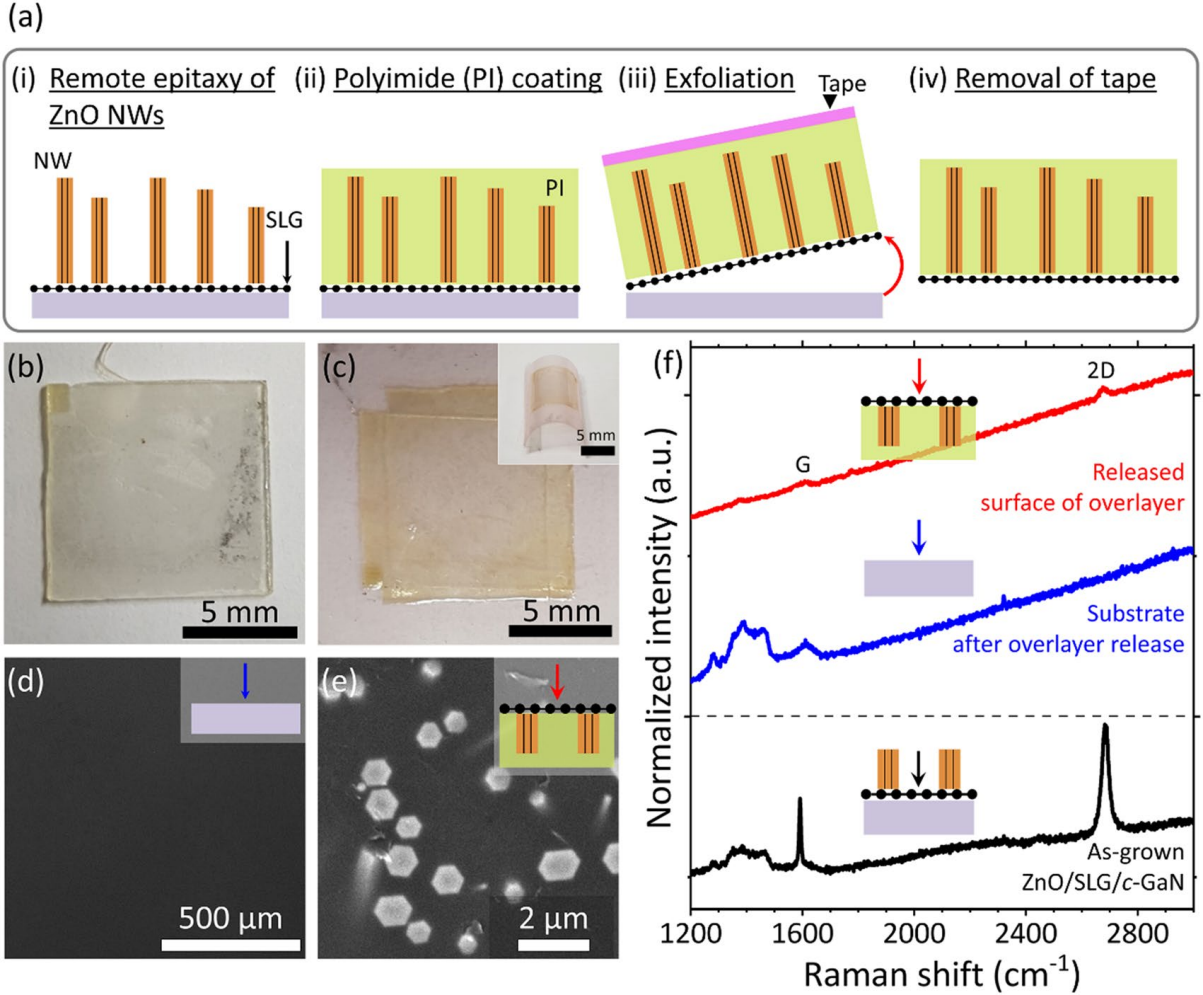
Exfoliation of remote epitaxial ZnO crystals using a sticky tape. (**a**) Illustration of procedures of exfoliation of ZnO crystals using thermal release tape. Photographs of (**b**) host substrate and (**c**) NWs-exfoliated thermal release tape after the exfoliation process. SEM images of (**d**) remained mother substrate and (**e**) bottom side of NWs-encapsulated PI film after the delamination. (**f**) Raman spectra of as-grown ZnO NWs/SLG/*c*-GaN before exfoliation (black line), the donor substrate after release (blue line), and the NWs-encapsulated PI film (red line) after exfoliation. Color arrows of the schematics represent the observed specimen surface with the direction of electron beam and laser irradiation in the SEM and Raman spectroscopic analyses, respectively.

The weakly bound interface of remote epitaxial ZnO/SLG/GaN allowed facile delamination of the overlayer ZnO crystals using a sticky tape. Before the exfoliation, the ZnO overlayer structures were spin-coated by PI, whose encapsulated film form of PI/ZnO crystals enabled to mechanically support and maintain the geometry of randomly grown arrays as they were. The sample of ZnO NWs with high *h*/*d* ratio was chosen for the delamination to validate the easy exfoliation. Thermal release tape was used to separate the PI/ZnO film from the original donor substrate, as illustrated in Fig. 7a. Figure 7b, c are the photographs of host substrate and PI/ZnO NWs-adhered thermal release tape after the delamination, respectively. The corresponding SEM images of Fig. 7d,e display surface of GaN mother substrate and bottom of PI/ZnO NWs peeled off from the substrate. Especially, as shown in Fig. 7e, although the delamination was performed by handwork, the verticality and homogeneous in-plane alignments of NWs were well maintained in the delaminated film of PI/ZnO NWs. This strongly suggests that the geometry and arrangement of epitaxial overlayer structures can be stamp-and-printed to the foreign surface without ruining them. Furthermore, the surface of substrate was smooth and clean without remaining residues after the delamination (Fig. 7d), which enabled us to re-use the substrate for the following remote epitaxy^8^. The existence of graphene was investigated by measuring the Raman spectra of as-grown ZnO NWs/SLG/GaN, the substrate after delamination, and exfoliated PI/ZnO samples (Fig. 7f). Graphene-related Raman shifts of G and 2D were observed from the as-grown ZnO NWs/SLG/GaN sample at 1,587 and 2,685 cm^–1^, respectively^67^. After the delamination, the peaks almost disappeared on the substrate, while the G and 2D peaks were still observed on the released surface of PI-encapsulated ZnO NWs. This indicates that the SLG was peeled off together with the PI/ZnO NWs overlayer during the exfoliation process. As for the delamination and Raman spectroscopic analysis of the other microcrystals, the same results were obtained from the MDs, MRs, and MNs. The Raman spectroscopic data of the MDs, MRs, and MNs are displayed in the Supplementary Materials (Fig. S7).

## Conclusion

Morphology-controlled remote epitaxy of ZnO micro- and nanostructures was demonstrated via hydrothermal synthesis. Four different types of ZnO morphologies (i.e., NW, MN, MR, and MD) were selectively synthesized in a controlled manner by choosing different anion type additives in the same nutrient solution. The electrostatic interaction between additives and charged facet surface was the key to control the facet-selective morphology control. The AR-STEM showed the remote heteroepitaxial relationship between ZnO and GaN substrate without polarity inversion across sub-nanometer gap of SLG. Furthermore, the as-synthesized ZnO micro- and nanostructures presented high optical quality of sharp NBE PL peak at low temperature. The non-covalent heterointerface of ZnO/SLG/GaN was utilized to peel the ZnO overlayer off from donor substrate by thermal release tape-assisted delamination technique without etching or congruent melting a sacrificial layer. We believe that the versatility of the wet chemical-based remote epitaxy for creating releasable, facet-controlled ZnO would be utilized in fabricating practical functional devices that have deformability and transferability.

## Supplementary Information

Supplementary Information.
