# Supplementary material for: Facet-selective morphology-controlled remote epitaxy of ZnO microcrystals via wet chemical synthesis
Source: Sci Rep. 2021 Nov 22;11:22697. doi: 10.1038/s41598-021-02222-1 (PMC8608950; doi:10.1038/s41598-021-02222-1)
Supplement: Supplementary file 1 — Supplementary Information. [file 41598_2021_2222_MOESM1_ESM.docx]

Supplementary Materials for

**Facet-selective morphology-controlled remote epitaxy of ZnO microcrystals via wet chemical synthesis**

Joonghoon Choi^1,2^, Dae Kwon Jin^1,2^, Junseok Jeong^1,2^, Bong Kyun Kang^3,4^, Woo Seok Yang^3^, Asad Ali^5^, Jinkyoung Yoo^6^, Moon J. Kim^7^, Gyu-Chul Yi^5^, & Young Joon Hong^1,2^

^1^Department of Nanotechnology and Advanced Materials Engineering, Sejong University, Seoul 05006, Republic of Korea

^2^GRI–TPC International Research Center, Sejong University, Seoul 05006, Republic of Korea

^3^Nano Materials Research Center, Korea Electronics Technology Institute (KETI), Seongnam, Gyeonggi-do 13509, Republic of Korea

^4^Department of Electronic Materials and Devices Engineering, Department of Display Materials Engineering, Soonchunhyang University, Asan, Chungnam 31538, Republic of Korea

^5^Department of Physics and Astronomy, Institute of Applied Physics, Seoul National University, Seoul 151-747, Republic of Korea

^6^Center for Integrated Nanotechnologies (CINT), Los Alamos National Laboratory, Los Alamos, NM 87545, United States

^7^Department of Materials Science & Engineering, The University of Texas at Dallas, Richardson, TX 75080, United States

*Corresponding author. Email: yjhong@sejong.ac.kr (Y.J.H.)

**The PDF file includes:**

Fig S1. Optical micrograph of CVD-grown graphene before complete coalescence.

Fig S2. Microcrystals grown by equimolar 15.0 mM solutions.

Fig S3. Multiple steps observed in tapered MN structure.

Fig S4. The effect of HCl in growth of MRs.

Fig. S5. Reproducibility of ZnO MRs synthesis.

Fig S6. Free exciton transition PL peak observed from ZnO MNs.

Fig S7. Raman spectra of as-grown ZnO microcrystals/SLG/c-GaN before exfoliation, the donor substrate after release, and the PI-encapsulated ZnO microcrystals film after exfoliation.

Table S1. Chemicals used for precursors and additives and the purity information.

Table S2. Solutions used for the ZnO microcrystals synthesis.


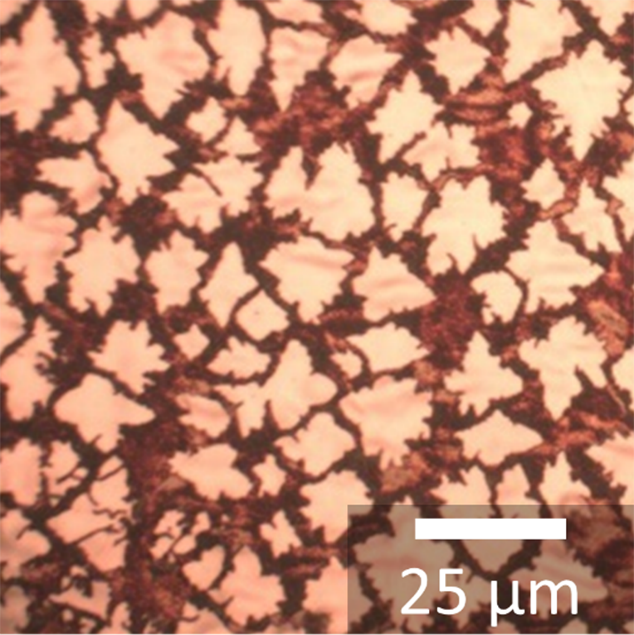


**Figure S1.** **Optical micrograph of CVD-grown graphene before complete coalescence.** Typical graphene domain size was measured at 5–20 μm. To estimate the graphene domain size, the growth was performed for 2 min before completion of coalescence. The complete coalescence can be achieved by growth time of at least of 10 min.


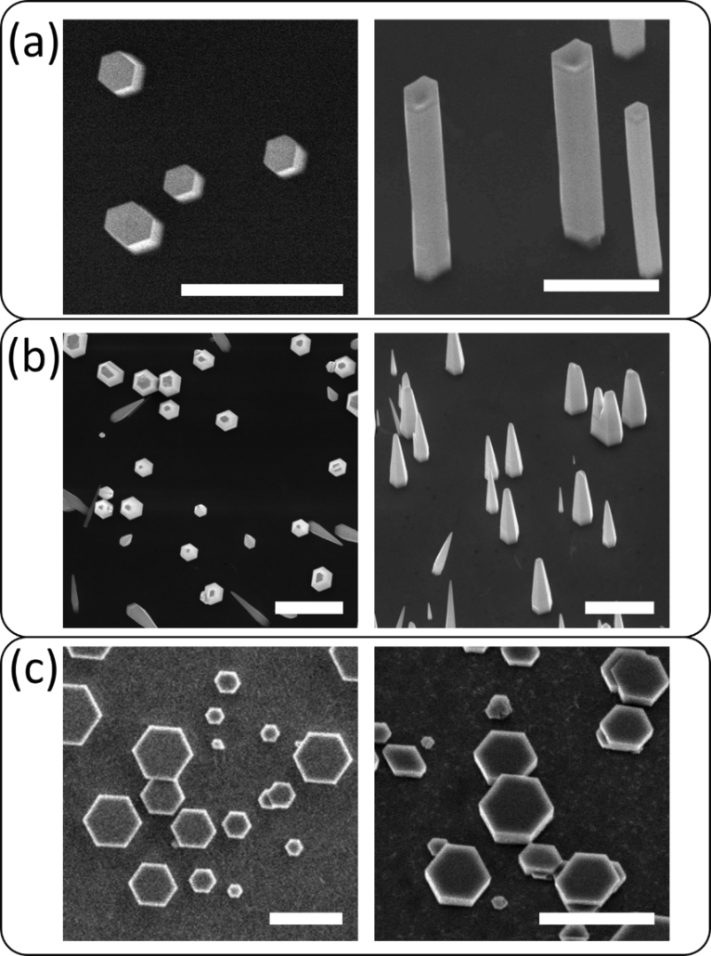


**Figure S2. Microcrystals grown by equimolar 15.0 mM solutions.** SEM images of ZnO (a) NWs, (b) MNs, and (c) MDs grown in the nutrient solutions of equimolar concentration 15.0 mM. Left and right images are top-view and tilt-view images, respectively. All the scale bars are 5 μm.


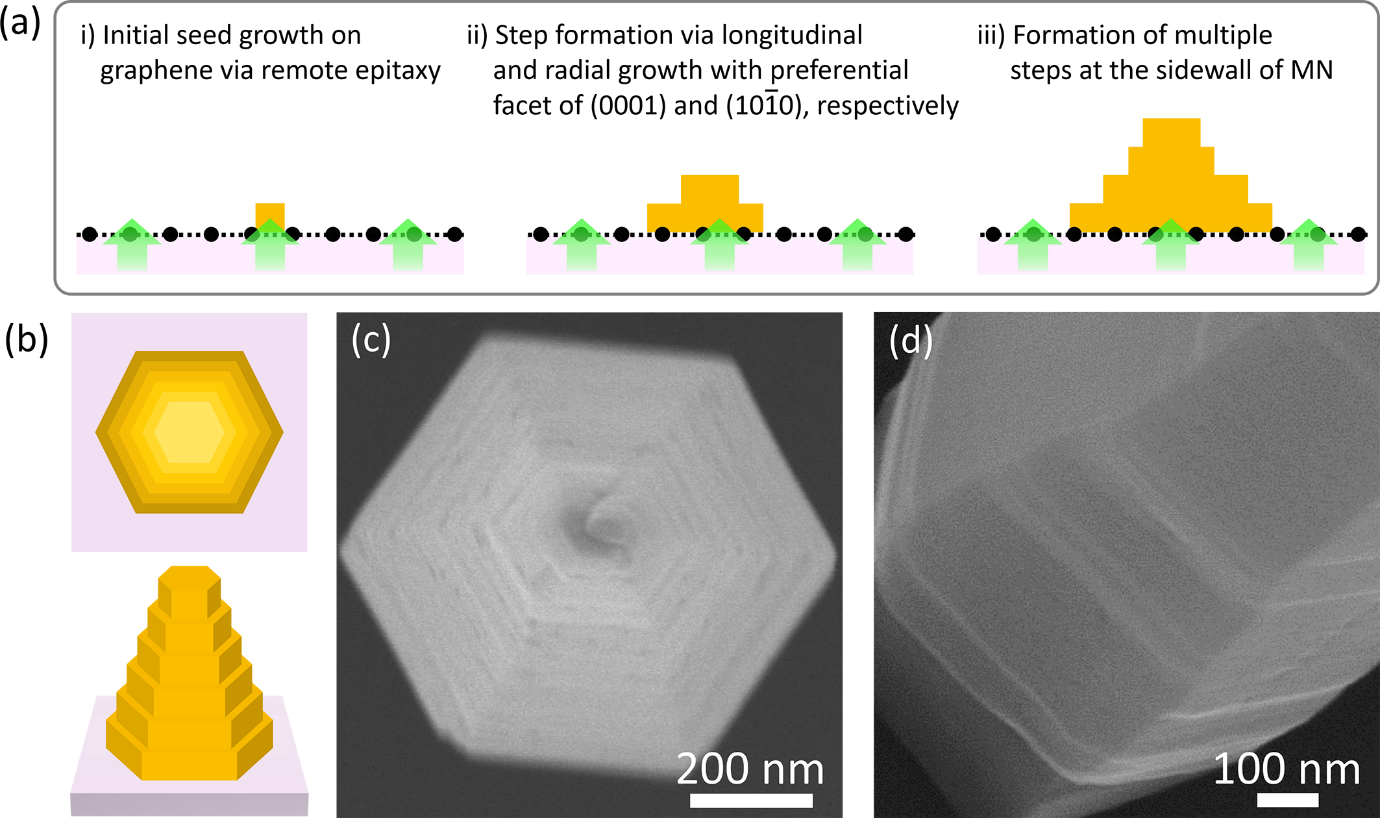


**Figure S3.** **Multiple steps observed in tapered MN structure.** (a) Illustrations depicting how the multiple steps form at the sidewall of during growth procedures. (b) Schematics of ZnO MN with multiple steps at the sidewall. (c) top-view and (d) tilted-view FE-SEM images of ZnO MN.


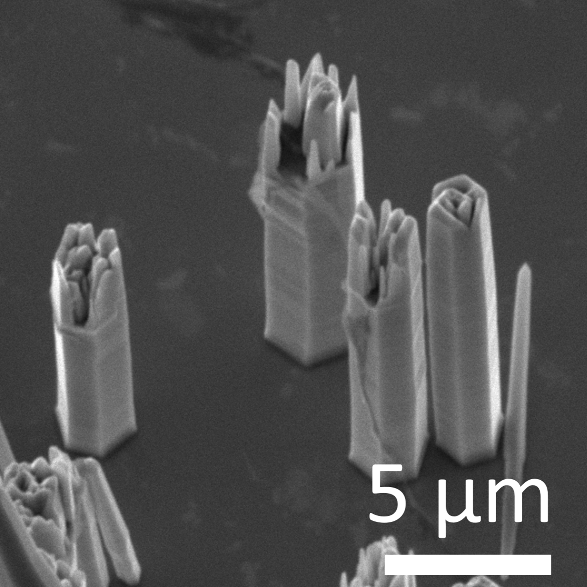


**Figure S4. The effect of HCl in growth of MRs.** Tilt-view SEM image of ZnO MRs grown in a nutrient solution at higher HCl concentration of 15.0 mM which was higher than the typical concentration of 8.8 mM for growing facet-smooth MRs.


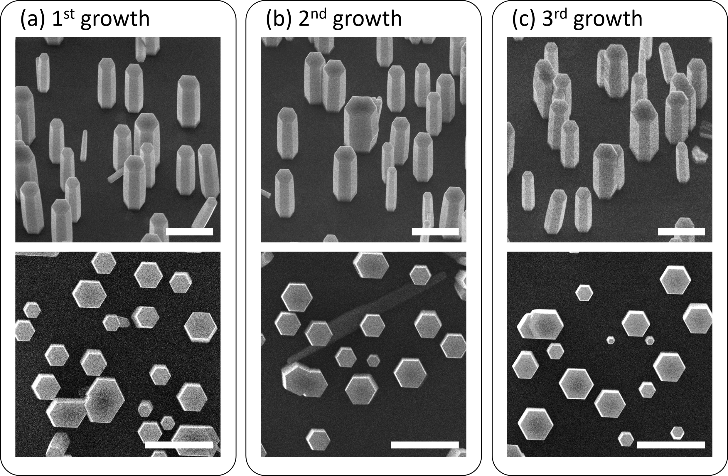


**Figure S5. Reproducibility of ZnO MRs synthesis.** (a–c) Tilt- and top-view SEM images of ZnO MRs obtained from different batches of the same nutrient solution condition. The reproducibility test was performed thrice in different days. All the scale bars are 5 μm.


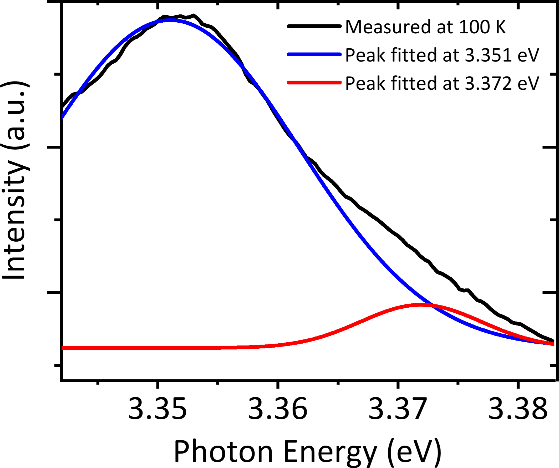


**Figure S6.** **Free exciton transition PL peak observed from ZnO MNs.** PL analysis for NBE emission of MNs. The NBE PL peak of MNs, measured at 100 K, was deconvoluted to separate into two PL peaks of neutral donor bound exciton and free exciton at 3.351 and 3.372 eV, respectively, via the multipeak analysis method.


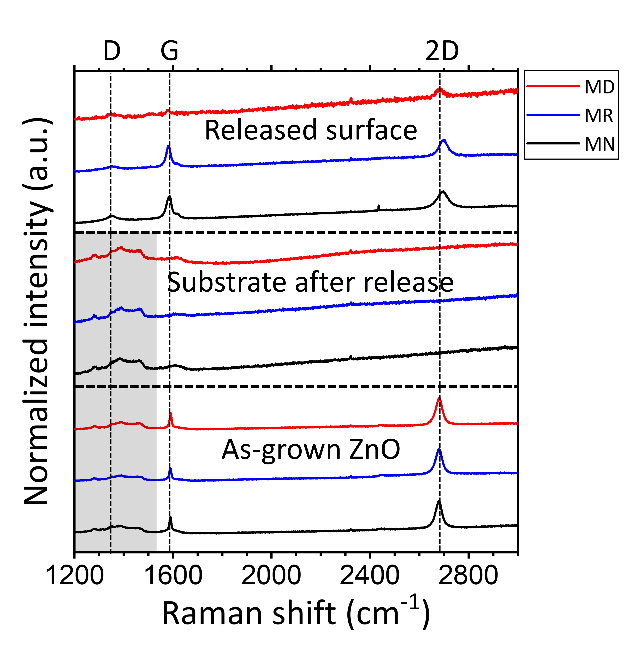


**Figure S7.** **Raman spectra of as-grown ZnO microcrystals/SLG/*c*-GaN before exfoliation (bottom panel), the donor substrate after release (middle), and the PI-encapsulated ZnO microcrystals film after exfoliation (top).** The MDs, MRs, and MNs are represented by black, red, and blue lines, respectively. The grey marked region shows the Raman shifts from GaN substrate, and positions of D, G, and 2D peaks are marked with vertical dot lines.

**Table S1. Chemicals used for precursors and additives
and the purity information**

| Precursors and additives | Purity |
| --- | --- |
| Zinc acetate dihydrate (ZAD) | ≥98% |
| Zinc nitrate hexahydrate (ZNH) | 98% |
| Hexamethylenetetramine (HMTA) | ≥99% |
| Trisodium citrate dihydrate (TCD) | 99% |
| Hydrochloric acid (HCl) | 33–34 % |

**Table S2. Solutions used for the ZnO microcrystals synthesis**

| Morphology | Nutrient solution | Additive |
| --- | --- | --- |
| NWs | DI water+ZAD+HMTA | – |
| MNs | DI water+ZNH+HMTA | – |
| MRs | DI water+ZNH+HMTA | HCl |
| MDs | DI water+ZNH+HMTA | TCD |
